# FluTyper-an algorithm for automated typing and subtyping of the influenza virus from high resolution mass spectral data

**DOI:** 10.1186/1471-2105-11-266

**Published:** 2010-05-19

**Authors:** Jason WH Wong, Alexander B Schwahn, Kevin M Downard

**Affiliations:** 1 Prince of Wales Clinical School & Lowy Cancer Research Centre, Faculty of Medicine, University of New South Wales, Sydney, NSW, Australia; 2 School of Molecular Science, University of Sydney, NSW, Australia

## Abstract

**Background:**

High resolution mass spectrometry has been employed to rapidly and accurately type and subtype influenza viruses. The detection of signature peptides with unique theoretical masses enables the unequivocal assignment of the type and subtype of a given strain. This analysis has, to date, required the manual inspection of mass spectra of whole virus and antigen digests.

**Results:**

A computer algorithm, FluTyper, has been designed and implemented to achieve the automated analysis of MALDI mass spectra recorded for proteolytic digests of the whole influenza virus and antigens. FluTyper incorporates the use of established signature peptides and newly developed naïve Bayes classifiers for four common influenza antigens, hemagglutinin, neuraminidase, nucleoprotein, and matrix protein 1, to type and subtype the influenza virus based on their detection within proteolytic peptide mass maps. Theoretical and experimental testing of the classifiers demonstrates their applicability at protein coverage rates normally achievable in mass mapping experiments. The application of FluTyper to whole virus and antigen digests of a range of different strains of the influenza virus is demonstrated.

**Conclusions:**

FluTyper algorithm facilitates the rapid and automated typing and subtyping of the influenza virus from mass spectral data. The newly developed naïve Bayes classifiers increase the confidence of influenza virus subtyping, especially where signature peptides are not detected. FluTyper is expected to popularize the use of mass spectrometry to characterize influenza viruses.

## Background

Influenza is a leading cause of death throughout the developed world and contributes to between 250,000 and 500,000 deaths every year worldwide [1]. On three occasions last century, global pandemics resulted in millions of deaths while recent pandemic threats have been posed by strains of avian [2] and swine origin [3]. Much higher rates of infection exist in the general population that, while not life threatening, inflicts illness and suffering. The virus also imposes a significant social and economic burden through productive losses in the workplace [4].

The genetic analysis of the influenza virus is derived from RT-PCR sequencing of amplified gene segments for the major antigens of the virus [5]. Most work is focused on the hemagglutinin gene because of its primary role in antigenic drift [6]. This is aided by the Influenza Virus Resource, a sequence database developed by the National Center for Biotechnology Information (NCBI) [7] that provides access to genetic sequence data that facilitates multiple sequence alignments, phylogenetic analysis and the generation of clusters [8,9]. It is typical in a retrospective analysis, for a strain from the most dominant genetic cluster within one influenza season to be recommended by the WHO for the vaccine in the following season.

Antigenic change is measured primarily employing the hemagglutination inhibition (HI) assay [10], where anti sera raised from infection of a host with one strain are cross reacted with other uncharacterized and reference strains in parallel. New computational approaches have been developed to analyze HI data [11] that increases the reliability with which antigenic differences can be assessed and this has been aided by mass spectrometric approaches [12] that enable epitopic domains to be localized [13-17]. Antigenic maps allow for the visualization of antigenic relationships among many strains in order to follow the short and long evolution of the virus [18]. These maps can aid the comparison of antigenic data derived from different laboratories and enable such data to be more reliably interpreted. Epidemiological modeling to predict whether new emerging strains are likely to cause widespread epidemics in future seasons is also under development [19,20]. The inclusion of antigenic drift and cross-immunity data can improve the reliability of these models.

We have recently developed the most direct and rapid method yet to survey influenza from the perspective of the viral protein antigens [21-24]. Antigens recovered from the virus or present in whole virus or vaccine preparations are digested with site-specific proteases and the peptide products are analyzed by high resolution mass spectrometry [25]. The mass accuracy attained in these analyzes enables the unambiguous identification of conserved signature peptides that are specific to a given type or subtype of the influenza virus. The signature peptides are unique in mass when compared to the *in silico *digest of all influenza proteins across all strains and hosts and those proteins known to contaminate virus preparations.

To date, the analysis of high resolution mass spectra of influenza proteolytic preparations has required manual interpretation through the identification of signature peptide masses that indicate the type or the subtype of an influenza virus. Currently, manual interpretation can be performed when signature peptides dominate a mass spectrum but it is not possible to establish the degree of confidence in typing and subtyping strains. Further, spectral analysis often involves the detection of multiple signature peptides, some of low abundance, or in some cases establishing the type and subtype without signature peptides (Po > 90-95). Existing algorithms such as the Mascot Peptide Mass Fingerprinting algorithm [26] can be used to identify proteins within a mass spectrum, however, such algorithms do not provide any level of confidence for the type and subtype of the virus from which the proteins are identified. This is particularly a problem when signature peptides are not detected in a given mass spectrum. To extend our previous work and automate the analysis of high resolution mass spectra of influenza proteolytic preparations, the FluTyper algorithm has been developed. FluTyper implements methods to deisotope, filter and detect peaks from mass spectra. Peaks are then matched against established signature peptides from common antigens [21-24]. In addition, naïve Bayes classifiers have been developed to provide statistical confidence for type and subtype assignments where few or no signature peptides are available. Here the basis of the FluTyper algorithm is described and its application for the automated analysis of MALDI mass spectra derived from antigen and whole virus digests is demonstrated.

## Results and Discussion

### Algorithm overview

**Figure 1:**
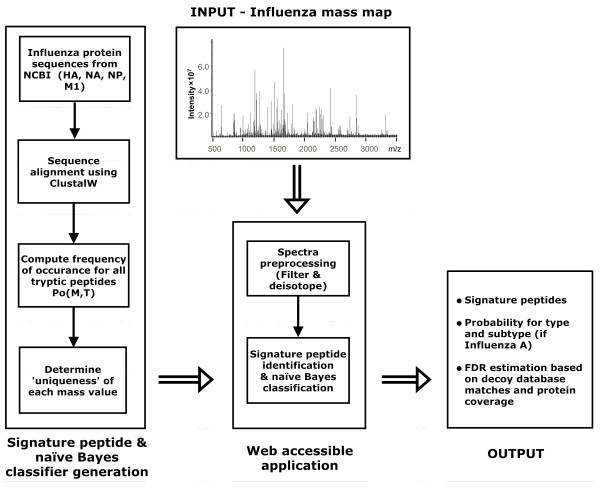
**Schematic overview of the FluTyper algorithm**.

FluTyper has been designed to utilize naïve Bayes classifiers for the typing and subtyping of proteolytic influenza mass spectra. FluTyper is divided into two main parts, first, the algorithm generates naïve Bayes classifiers and determines unique signature peptides, and second, the algorithm pre-processes query mass spectra and determines the virus type and subtype based using the classifiers and signature peptides (Figure 1). Naïve Bayes classifiers are generated for four common influenza antigens hemagglutinin (HA), neuraminidase (NA), nucleoprotein (NP), and matrix protein 1 (M1). Subsequently, the FluTyper algorithm uses all classifiers, in combination, for the computation of the type and subtype probabilities and the identification of proteolytic signature peptides from each mass spectrum analyzed.

### Pre-processing of high resolution mass spectra

Mass spectra of tryptic influenza peptides are pre-processed prior to typing and subtyping using the naïve Bayes classifier. First, a user defined threshold is used to remove peaks that are considered to be noise (typically set at a signal-to-noise ratio of 2). Second, all isotope clusters are identified and the spectrum is deisotoped. The deisotoping method used is adapted from the THRASH algorithm [27]. The method involves iterating through each peak in the threshold mass spectrum starting from the lowest *m/z *value. As the algorithm proceeds, each peak is compared to previous peaks to determine if it belongs to an existing isotopic cluster. If a peak belongs to an existing isotopic cluster, the peak is removed and its intensity is added to the existing monoisotopic peak. To evaluate the composition of isotopic clusters, the model amino acid *averagine *(C_4.9384_H_7.7583_N_1.3577_O_1.4773_S_0.0417_) [28] is used to define both the predicted distance between isotopic peaks and the intensity distribution of ions with an isotopic cluster. A major advantage of mass spectral data acquired by MALDI is that tryptic peptide ions generated are almost exclusively singly charged (i.e. [M+H^+^] ions). This eliminates the need to deconvolute (by mass) the mass spectrum.

### Naïve Bayes classifiers for the typing and subtyping of the influenza virus

Non-redundant HA, NA, NP and M1 sequence sets for human strains of influenza virus type A and B, and subtypes H1N1 excluding pandemic sequences (H1N1) 2009 sequences, pandemic (H1N1) 2009 sequences (P2009), H3N2 and H5N1 were retrieved from the NCBI Influenza Virus Sequence Database [7]. Each set of sequences is then aligned using ClustalW [29] to enable the relative frequency of occurrence *Po(M, T) *of each unique theoretical monoisotopic tryptic peptide ion [M+H]^+^, *M*, for a given type or subtype, T, to be determined. Tryptic peptide fragments were generated to allow for up to 2 missed cleavages, with fixed carbamidomethyl cysteine and optional modifications of methionine, glutamic acid and cysteine residues in the form of oxidized methionine, pyroglutamate and acrylamide adducts with cysteine.

A naive Bayes classifier is a simple probabilistic classifier based on the application of Bayes' theorem. Using the classifier, the type or subtype of an influenza virus can be determined as follows:(1)

where *p(T|M_1_...M_n _) *is the probability for a type or subtype T based on theoretical tryptic peptide ion monoisotopic masses, *M_1_...M_n_*. All parameters (*p(M_i_|T*), *p(T) *and *p(M_1_...M_n_)*) in the model are estimated directly from protein sequence alignments. The independent probability for each mass to be present for a given type or subtype, *p(M_i_|T)*, is given by its relative frequency of occurrence *Po(M, T)*. The assumption is made that the presence of peptide ion masses derived from a particular protein is independent to that of any other mass (i.e. that the presence of one tryptic peptide is independent of the presence of another). Where a particular mass *M_i _*is present in one type or subtype, but not another, the Laplace's rule of succession is applied, where 1 is added to the number of observed events to avoid zero probabilities. This assumption is useful to account for noise peaks that may be present in mass spectral data. The prior probability, *p(T)*, reflects the probability of occurrence of a given type or subtype, T, and is estimated based on the relative number of sequences in the NCBI database for T. However, this value may be adjusted as necessary to match the observed occurrence of different influenza types and subtypes in a particular season. Finally, the independent probability of observing peaks *M_1_...M_n_*, *p(M_1_...M_n_) *can be computed as the sum of the probability of observing peaks *M_1_...M_n _*across all types or subtypes:(2)

where *T_a_*, *T_b_*, *T_x_*, etc are all the possible type or subtypes being analyzed. A naïve Bayes classifier is built for each of the HA, NA, M1 and NP antigens used to type and subtype the virus.

To assess the peak matching false discovery rate, decoy naïve Bayes classifier models are generated using randomly permutated sequences from the same set of influenza proteins.

### Uniqueness of peptide ion masses in naïve Bayes classifiers

Since the naïve Bayes classifier is trained based on theoretical protein sequences from specific influenza proteins alone, validation that the tryptic peptide masses are unique to influenza is necessary. This is performed as described previously [21]. Briefly, each theoretical monoisotopic mass, M, from each type and subtype present in the naïve Bayes classifier, is compared against the theoretical monoisotopic tryptic ion masses [M+H^+^] from a custom database containing all non-redundant influenza protein sequences, and those of possible contaminants, including human keratin, bovine/porcine trypsin and several chicken proteins that have been found to commonly contaminate egg-propagated virus preparations or are introduced during the sample preparation. The included egg-derived chicken protein contaminants are based on our own observation and their identity was confirmed by MALDI tandem mass spectrometry (unpublished observations - spectra available upon request). Other unknown contaminants are always possible, but due to the use of high-resolution mass spectrometry with mass accuracies routinely better than 1 ppm achieved, the misassignment of contaminants will be largely avoided. Masses are generated for predicted tryptic peptide ions allowing for up to 2 missed cleavages and the same post-translational modifications as described in the previous section. The difference in M and the closest theoretical mass, *U_M _*(in parts per million (ppm)), of a tryptic peptide derived from a contaminant or influenza antigen with at least 10 entries in the custom database is defined as the uniqueness.

### Peak matching, signature peptide identification and computation of type and subtype probabilities using naïve Bayes classifiers

In a mass spectrum, typically only a portion of theoretical tryptic peptides is observed experimentally. This may be due to a range of factors ranging from incomplete proteolytic cleavage to the presence of unanticipated post-translational modifications. It is necessary to first define a set of theoretical tryptic peptide masses that are actually observed within a specified mass error tolerance. The list of theoretical masses used for matching are determined based on the specified protein (HA, NA, NP, M1 or all). Where the mass of an observed peak is within the mass error tolerance of two or more peaks, the closest theoretical mass is selected. For a matching peak to be selected for further analysis, the mass must be sufficient unique as defined by:(3)

where *ΔM *is the mass error (in ppm) between the observed mass and theoretical tryptic peptide mass, and *U_M _*is the uniqueness as described in the previous section. A scaling of *U_M _*by a factor of 0.5 is necessary to ensure that there cannot be another tryptic contaminant peptide mass present that is closer to the observed mass than that of the theoretical mass.

The concept of using signature peptides to type and subtype the influenza virus has been previously described [21]. A signature peptide is defined as a theoretical tryptic peptide that is exclusively present in one type or subtype, but not in any of the others. In the FluTyper algorithm, a signature peptide is defined as any theoretical tryptic peptide, M, where *Po(M, T) *> 0.7 for one type or subtype and *Po(M, T) *= 0 for all other types or subtypes for a given influenza protein. Since few signature peptides may be indicative of a particular subtype of the virus, indicator peptides are also used by the algorithm. An indicator peptide is defined similarly to a signature peptide with the exception that it may occur in the sequence of antigens from other viral subtypes with *Po(M, T) *< 0.1.

For the computation of type and subtype probabilities, the naïve Bayes classifier (1) is applied using the set of matching peaks. For typing, this provides a probability that a set of masses is from influenza A (p(*FluA| M_1_...M_n_*)) or influenza B (p(*FluB|M_1_...M_n_*)). If p(*FluA|M_1_...M_n_*) > 0.7 or there is more than one influenza A signature peptide identified, the algorithm will proceed to perform subtyping where p(*H1N1|M_1_...M_n_*), p(*H3N2|M_1_...M_n_*), p(*H5N1|M_1_...M_n_*) and p(*P2009|M_1_...M_n_*) are all computed.

### Implementation

Since it is only necessary to generate a naïve Bayes classifier when new sequences have been added to the custom database, the implementation of the FluTyper algorithm is divided in two applications, consisting of the naïve Bayes classifier and signature peptide generator, and the mass spectrum analysis program (Figure 1). The classifier and signature peptide generator accepts ClustalW aligned sequences as input to compute the frequency of occurrence of theoretical tryptic peptides and determines the uniqueness of their mass. The output is a table containing all data necessary for naïve Bayes classification and signature peptide determination. The second component of FluTyper accepts a mass spectrum in ASCII format and the classification tables as input. FluTyper outputs the type and subtype prediction based on signature peptides and naïve Bayes probabilities. The number of matches to peptides from decoy sequences is also shown to provide an estimate of the false positive peak matching rate. A summary of all peaks identified can also be downloaded in tab-delimited format. FluTyper is implemented using GNU C++. A web interface has been developed for the second component of FluTyper and can be accessed at http://www.cancerresearch.unsw.edu.au/CRCWeb.nsf/page/flutyper (see Figure S1 for a screenshot of the interface and Table S1 for a description of the parameters).

### Theoretical evaluation of naïve Bayes classifier

The performance of the naïve Bayes classifiers were evaluated as a function of the protein coverage. For each protein (i.e. HA, NA, NP or M1), 500 random subsets of theoretical tryptic peptides representing 0-100% coverage of the protein were generated for each protein sequence used to train the classifier. The set of theoretical tryptic peptides masses represents a simulated mass spectrum. Leave-one-out cross-validation was performed, meaning that a new classifier was used each time, leaving out the protein sequence being tested. For the purpose of this evaluation, a subset of masses were determined to be typed or subtyped if *p(T| M_1_...M_n_) *> 0.7 for any T.

**Figure 2:**
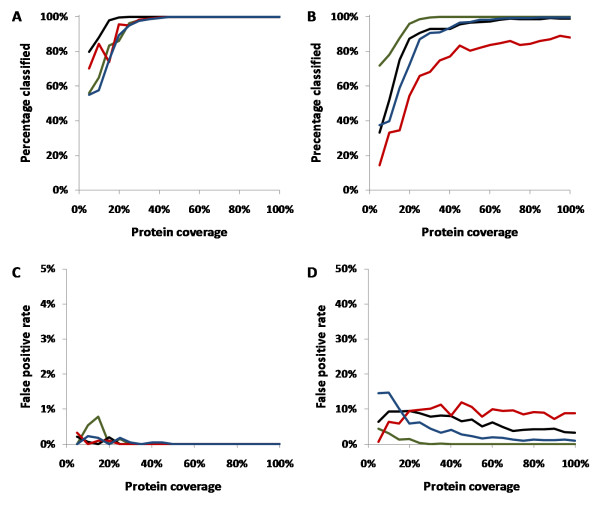
**Performance of naïve Bayes classifier relative to protein coverage in terms of generating conclusive results for typing (A) and subtyping (B) of the influenza virus and in terms of false positive rates for typing (C) and subtyping (D).** In all subfigures, the black, red, green and blue lines represent NP, M1, HA and NA respectively.

Figure 2A &2B shows the percentage of simulated mass spectra conclusively classified as a function of protein coverage for typing and subtyping respectively. For typing, over 90% classification rate was achieved with greater than 25% protein coverage in all cases. For subtyping, over 90% classification rate was achieved with greater than 30% protein coverage for HA, NA and NP. However, M1 was less reliable, with a classification rate limited to around 80% with a protein coverage of greater than 40%. The low classification rate for M1 is due to a combination of factors. First, the M1 protein has around 50% less amino acids compared to NP, NA and HA and therefore also has fewer tryptic peptide masses that can be used by the naïve Bayes classifier. Second, the M1 protein is more conserved between different influenza subtypes compared to NP, NA and HA, thus the classifier may not be able distinguish the subtype even with full protein coverage.

In the case of typing (Figure 1C), the false positive rate (FPR) is less than 1% in all cases and 0% at protein coverage of greater than 25%. For subtyping (Figure 1D), the FPR was less than 1% for protein coverage of 20% or greater for HA and less than 5% with increased sequence coverage for NA. HA performed more favorably than NA since the neuraminidase of H1N1 and H5N1 are similar, while the hemaggluttin antigen across H1N1, H3N2 and H5N1 are all significantly different. On the other hand, the NA classifier was able to distinguish H*x*N1 and H3N2 subtypes with 0% FPR (data not shown).

For NP, the FPR is 10% at low protein coverage and decreases to 5% with increased coverage. For M1, the FPR is just under 10% independent of the protein coverage. The high apparent FPR for NP and M1 for subtyping can be expected since the subtype of a virus is characterized by the isoform of its HA and NA proteins. For instance, the reassortment of a virus can lead to the introduction of a NP protein from one subtype to another (e.g. H1N1 to H3N2) without changing the subtype of the actual virus. For example, the translated NP protein sequence derived from the NCBI entry gi148466309 is designated as a H3N2 subtype, but the actual sequence is in fact more similar to other H1N1 NP sequences.

The theoretical testing results demonstrate that the use of naïve Bayes classifiers are appropriate at protein coverage levels expected from experimental mass spectra where 20-30% or greater protein coverage is common. Crucially, the false positive rate is less than 1% for typing and is still below 10% for subtyping using M1 and NP proteins. It is evident from testing that for confident assignment of the virus subtype, the use of HA or NA tryptic peptides would be most desirable.

### Testing with experimental influenza mass spectra

To demonstrate FluTyper using experimental data, mass spectra were acquired from tryptic digests prepared from whole virus preparations and gel-separated influenza antigens. Mass spectra were generated for common human influenza virus strains including influenza type B strain B/Victoria/504/2000, type A (H1N1) strain A/Solomon Islands/03/06 and type A (H3N2) strain A/Brisbane/10/2007 (Additional file 1). The type and subtype of these three strains are in common with those viruses that are in circulation in humans today. All samples were analyzed using default FluTyper settings - with relative peak intensity cutoff at 0.001%, peak matching tolerance of 3 ppm, frequency of occurrence (Po) cutoff of 0.6, one missed cleavage and optional modification of methionine oxidation.

**Figure 3:**
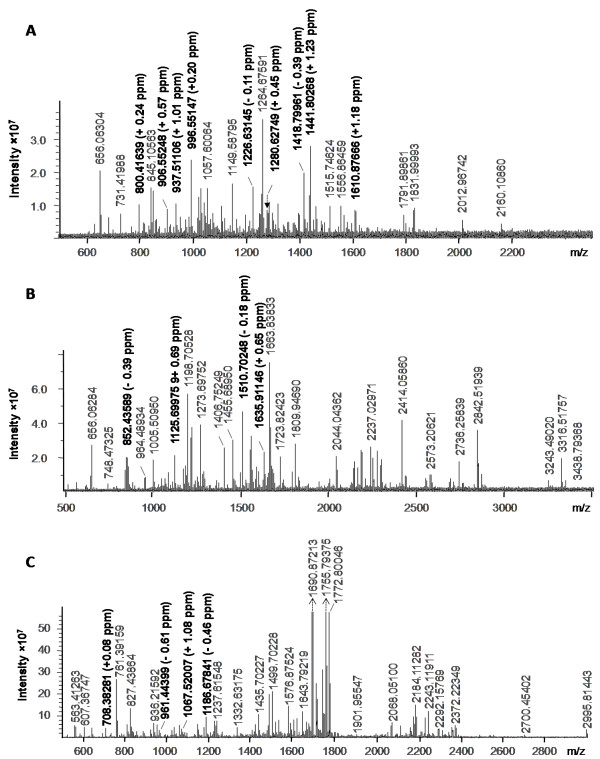
**High resolution mass spectrum of (A) whole virus digest of influenza type B strain B/Victoria/504/2000, (B) whole virus tryptic digest of influenza type A (H3N2) strain A/Brisbane/10/2007, and (C) in-gel tryptic digest of the nucleoprotein from type A (H1N1) strain A/Solomon Islands/03/06**.

The high resolution mass spectrum of a whole virus digest of influenza type B strain B/Victoria/504/2000 is shown in Figure 3A. The 15 signature peptides for influenza type B identified enable the virus type to be confidently assigned (Table 1). In addition to the signatures, 3 indicator peptides - those that are present with a frequency of occurrence, Po < 0.1 in all other types, are also identified. The identified signature and indicator peptides are distributed amongst NP, M1, NA and HA, showing that good sequence coverage of all major antigens can be achieved through whole virus digestion.

**Table 1:** Identified peptides from a mass spectrum (Figure 3A) of a whole virus digest of type B influenza strain B/Victoria/504/2000

					Po			
Sequence	[M+H]^+^	Start-End	Δppm	Uniqueness(in ppm)	FluA	FluB	Protein	Type signature
GGFVHQR	800.41620	354-360	-0.2449	-1.6716	0.0000	**0.9962**	NA	*
SHFANLK	816.43626	69-75	0.2094	12.1210	0.0000	**0.7320**	HA	*
QLPNLLR	853.52541	136-142	-0.3046	-55.8003	0.0000	**0.9268**	HA	*
GLILAERK	899.56728	95-102	-0.9505	4.4710	0.0001	**0.8696**	M1	
TIYFSPIR	996.55129	163-170	-0.1806	3.3917	0.0001	**0.9504**	NP	
AGLNDDMER	1020.44149	97-105	0.3116	32.3536	0.0000	**1.0000**	NP	*
LQFWAPMTR	1149.58736	439-447	-0.5106	2.1677	0.0000	**1.0000**	NP	*
QTIPNFFFGR	1226.63167	540-549	0.1753	4.7871	0.0000	**0.9504**	NP	*
SMVVVRPSVASK	1259.71402	320-331	-0.3271	-11.0477	0.0000	**0.9587**	NP	*
LNVETDTAEIR	1260.64302	309-319	0.4815	-1.9918	0.0000	**0.9660**	NA	*
NLIQNAHAVER	1264.67566	106-116	-0.2001	-0.8042	0.0000	**0.9256**	NP	*
SKPYYTGEHAK	1280.62696	336-346	-0.4123	2.6393	0.0000	**0.9892**	HA	*
LGEFYNQMMVK	1359.64355	86-96	-1.3173	8.2625	0.0000	**0.9917**	NP	*
VLSALTGTEFKPR	1418.80019	399-411	0.4130	-6.9756	0.0000	**0.9752**	NP	*
LAEELQSNIGVLR	1441.80092	201-213	-1.2249	7.7910	0.0000	**0.9149**	M1	*
EFDLDSALEWIK	1465.72094	36-47	-0.2681	-2.7440	0.0000	**0.9574**	M1	*
REMQMVSAMNTAK	1496.70182	175-187	-0.3554	2.1066	0.0001	**0.9149**	M1	
NPGIADIEDLTLLAR	1610.87481	305-319	-1.1441	6.9732	0.0000	**0.9917**	NP	*

Naïve Bayes probabilities: P(*FluB*) = 1.000

To demonstrate the subtyping ability of FluTyper, a whole virus digest of type A (H3N2) influenza strain A/Brisbane/10/2007 is used (Figure 3B). In total, there are 18 peaks with Po of > 0.6 and the peaks are matched within the 3 ppm threshold (Table 2). 8 of the 18 peaks identified are signature peptides for type A influenza.

**Table 2:** Identified peptides from a mass spectrum (Figure 3B) of a whole virus digest of type A (H3N2) influenza strain A/Brisbane/10/2007

					Po						
Sequence	[M+H]^+^	Start-End	Δppm	Uniqueness (in ppm)	H1N1	**H3N2**	H5N1	P2009	Protein	Type signature	Subtype sig/ind
HENR	555.26338	175-178	0.0432	52.5444	1.0000	**0.9944**	1.0000	1.0000	M1	*	
NFWR	622.30961	211-214	0.2025	15.9037	1.0000	**0.9975**	0.9885	1.0000	NP	*	
RIWR	630.38344	124-127	0.7773	-2.1209	0.8377	**0.9687**	0.0235	0.0000	NP		
LLSFIR	748.47159	349-354	-2.2232	-15.0079	0.0319	**0.9171**	0.0000	0.0000	NP		*
SGYWAIR	852.43626	389-395	0.4282	-5.2567	0.0000	**0.7024**	0.0000	0.0000	NP		*
QMVQAMR	863.42261	211-217	0.9335	3.6413	0.9474	**0.9396**	0.9730	0.0000	M1	*	
EITFHGAK	902.47304	106-113	1.7441	3.7342	0.9794	**0.9670**	0.6944	0.9841	M1		
MVLSAFDER	1067.51901	72-80	1.1213	-6.9338	1.0000	**0.9974**	1.0000	0.9877	NP	*	
TRPILSPLTK	1125.69902	48-57	-0.6476	19.9583	1.0000	**0.9727**	1.0000	1.0000	M1	*	
GINDRNFWR	1177.58612	206-214	0.2675	-1.1430	0.8883	**0.8744**	0.0345	0.9726	NP		
MVLSAFDERR	1223.62012	72-81	-0.9578	5.3252	0.9628	**0.9844**	0.0000	0.9877	NP		
GIGTM*VMELIR	1235.64864	191-201	-0.5722	-2.7281	0.0106	**0.8241**	0.0116	0.0000	NP		
MMEGAKPEEVSFR	1510.70286	453-465	0.2529	2.2314	0.0053	**0.8317**	0.0000	0.0058	NP		
GWAFDDGNDVWMGR	1625.68015	357-370	-2.3418	79.7986	0.0000	**0.9116**	0.0000	0.0000	NA(N2)	*	*
GILGFVFTLTVPSER	1635.91047	58-72	-0.6046	-1.6425	0.9588	**0.9670**	0.8947	0.9831	M1	*	
NPGNAEIEDLIFLAR	1671.87006	253-267	0.8739	-6.7188	0.2606	**0.7281**	0.9892	0.9589	NP	*	
MMEGAKPEEVSFRGR	1723.82544	453-467	0.7002	-1.9602	0.0053	**0.6533**	0.0000	0.0000	NP		
ESRNPGNAEIEDLIFLAR	2044.04579	250-267	1.0631	-3.5273	0.2128	**0.7482**	0.0538	0.9315	NP		

Naïve Bayes probabilities: P(*FluA*) = 1.000, P(*H3N2*) = 1.000; M* indicates methionine sulfoxide

Generally, type signature peptides are highly conserved with Po > 0.95 across all subtypes and provide little value for distinguishing subtypes (this is with the exception of the NA peptide (1625.68015 m/z) which is only present in HxN2 sub-types). Nevertheless, of the remaining 10 peptides, FluTyper identified two as H3N2 subtype signatures (852.43626 m/z and 1625.68015 m/z) and one as an indicator (748.47159 m/z). The identification of the signature and indicator peptides alone enables the subtype to be confidently assigned to H3N2. Furthermore, by applying the naïve Bayes classifier using the Po values of all the peaks for all subtypes a *p(H3N2|peaks) *value of 1 is obtained, providing additional confidence of the result (see Additional files 2, 3, 4 and 5).

Finally, to demonstrate the use of the naïve Bayes classifier where no signature peptides are available for subtyping, a mass spectrum of in-gel digested nucleoprotein from type A (H1N1) strain A/Solomon Islands/03/06 was analyzed (Figure 3C). In total, 11 peptides are identified by FluTyper (Table 3). While 5 type A influenza signatures peptides are identified, no subtype indicator or signature peptides were found. In this case, the naïve Bayes classifier provides the only means for subtype determination. Using the Po values shown in Table 3, the classifier generates probabilities of 0.9998, 0.0002, 0 and 0 for H1N1, H3N2, H5N1 and P2009 respectively, indicating that the peptides identified are almost certain to have come from the H1N1 subtype.

**Table 3:** Identified peptides from a mass spectrum (Figure 3C) of nucleoprotein derived from type A (H1N1) influenza strain A/Solomon Islands/03/06

					Po						
Sequence	[M+H]^+^	Start-End	Δppm	**Uniqueness (in ppm)**	**H1N1**	H3N2	H5N1	P2009	Protein	Type signature	Subtype sig/ind
NFWR	622.30961	205-208	0.0578	15.9037	**1.0000**	0.9975	0.9885	1.0000	NP	*	
MIGGIGR	703.39196	32-38	0.0668	9.2751	**0.7249**	0.0078	0.0000	0.9877	NP		
YWAIR	708.38276	385-389	-0.0663	-16.7649	**1.0000**	0.2902	1.0000	1.0000	NP	*	
SRYWAIR	951.51590	383-389	1.4419	10.4002	**0.8095**	0.1878	0.8495	0.0411	NP		
SGGNTNQQR	961.44460	392-400	0.6313	3.6976	**0.8783**	0.9724	0.5161	0.0137	NP	*	
MVLSAFDER	1067.51901	66-74	-0.9958	-6.9338	**1.0000**	0.9974	1.0000	0.9877	NP	*	
M*VLSAFDER	1083.51392	66-74	-2.1209	-12.6763	**1.0000**	0.9974	1.0000	0.9877	NP		
MVLSAFDERR	1223.62012	66-75	2.0251	5.3252	**0.9628**	0.9844	0.0000	0.9877	NP		
FYIQMCTELK	1332.63265	45-54	0.6754	32.3082	**0.9865**	0.9948	0.9560	1.0000	NP	*	
SYEQM*ETDGER	1360.53214	9-19	1.6854	8.0895	**0.9096**	0.3526	0.0000	0.0260	NP		
M*CSLM*QGSTLPR	1412.63307	163-174	2.5130	-7.6276	**1.0000**	0.9974	0.9765	1.0000	NP		

Naïve Bayes probabilities: P(*FluA*) = 1.000, P(*H1N1*) = 0.9998; M* indicates methionine sulfoxide

To validate the naïve Bayes classification, the protein sequence coverage is shown in Table 4. In the case of the whole virus digests, a coverage range of between 10.5% and 42%, and 10.3% and 27.9% was achieved in mass spectra for the type A (H3N2) and type B virus, respectively. The combined FPR as estimated from Figure 2B and 2D based on the product of each of the individual antigen FPR is < 0.1% for type A (H3N2) and type B, respectively. For type A (H1N1), as expected, only nucleoprotein was identified for the in-gel digestion of this antigen with a sequence coverage of 24.8%. Based on theoretical testing from Figure 2D, there is an approximately 8% chance that the spectrum could be misidentified. As discussed earlier, the high false positive rate is due to the fact that the subtype of an influenza virus is defined based on hemagglutinin and neuraminidase, hence the possibility of reassortment cannot be excluded. Nevertheless, the nano-scale preparation and mass spectrometry analysis of whole virus digests described here provides highly reliable subtyping results for influenza using FluTyper.

**Table 4:** Total protein coverage of the different antigens identified from the mass spectrum of each of samples tested

	Protein coverage			
Sample	HA	NA	NP	M1
Influenza B (Victoria)	12.0%	10.3%	27.9%	18.5%
H3N2 (Brisbane)	19.3%	10.5%	42.0%	33.3%
H1N1 (Solomon Island)	n/a	n/a	24.8%	n/a

## Conclusions

The FluTyper algorithm has been developed for automated typing and subtyping of influenza virus using high resolution mass spectral data. FluTyper incorporates the use of influenza antigen signature peptides previously identified in this laboratory. Furthermore, to increase the confidence of subtyping, naïve Bayes classifiers have been developed for four common influenza antigens, hemagglutinin, neuraminidase, nucleoprotein, and matrix protein 1. Theoretical testing of the classifiers demonstrates their applicability at protein coverage rates expected in mass mapping experiments. Using laboratory grown virus samples analyzed by high resolution mass spectrometry, it is shown that FluTyper can rapidly and reliably type and subtype strains of the influenza viruses that are in common circulation in humans. Through the use of other signature peptides and classifiers, it is anticipated that the FluTyper algorithm could be applied to the typing/classification of other viruses and bacteria.

## Methods

### Influenza virus strains

All utilized human strains of type A and type B influenza viruses, A/Solomon Islands/03/06(H1N1), A/Brisbane/10/07(H3N2), and B/Victoria/504/2000, were purchased from Advanced ImmunoChemicals Inc. (Long Beach, California, USA). The inactivated viruses, prepared from allantoic fluid of embryonated eggs, were used without further purification.

### Protein preparation and digestion

A suspension corresponding to 35 μg of influenza virus type B and type A (H1N1), was evaporated to near dryness, resuspended in digestion buffer without trypsin (50 mM NH_4_HCO_3_, 10% ACN, 2 mM DTT) and incubated at 37°C for 3 h. Modified trypsin (1.0 mg•mL^-1^; Roche Diagnostics GmbH, Mannheim, Germany) was added to a final concentration of about 30 ng•μL^-1 ^and the digestion carried out at 37°C over night.

Where gel recovered, viral protein was first separated from 20 μg of the virus by SDS-PAGE (12.5%), excised and destained (25 mM NH_4_HCO_3 _in 50% acetonitrile). The reduction and alkylation of cysteine residues with DTT (10 mM DTT, 50 mM NH_4_HCO_3_; 30 min, 56°C) and iodoacetamide (55 mM iodoacetamide, 50 mM NH_4_HCO_3_; 20 min at room temperature in the dark) was followed by tryptic digestion as previously described [21]. Cleaved peptides were extracted by repeated sonication in 60% acetonitrile containing 0.1% trifluoroacetic acid. Extracted peptides were dried completely in a vacuum concentrator and dissolved in 25 mM NH_4_HCO_3_.

### Nano-scale digestion of whole virus

2.5 μL of a suspension containing 500 ng•μL^-1 ^of the influenza virus type A (H3N2) was irradiated in a microwave (Samsung MX245) at 900 W power for 2 × 20 s. 7.5 μL of a 2.6 mM DTT solution was added to reduce Cysteine residues. The sample was sonicated in a sonicator bath and incubated at 60°C in an Eppendorf thermomixer for 30 min. The suspension was evaporated to dryness in a vacuum concentrator and viral protein was reconstituted in 4 μL digestion buffer (31.3 mM NH_4_HCO_3_, 12.5% acetonitrile, 4.3 mM octyl-β-D-glucopyranoside) by vortexing and sonication. 1.0 μL modified trypsin (65 ng•μL^-1^; Roche Diagnostics, Mannheim, Germany) was added and the digestion carried out overnight at 37°C. The digestion mixture was concentrated to dryness and the tryptic cleavage products were dissolved directly in matrix solution (1.5 mg•mL^-1 ^α-cyano-4-hydroxycinnaminic acid, 6.3 mM NH_4_HCO_3_, 45% acetonitrile, 0.075% TFA) to create a peptide concentration of ~250 ng•μL^-1^.

### MALDI FT-ICR mass spectrometry

MALDI FT-ICR mass spectra were recorded on a 7T Bruker APEX-Qe instrument (Bruker Daltonics, Billerica, MA, USA) in the positive ion mode as previously described [21-24]. Briefly, mass spectra were acquired for 1 M data points using a broadband excitation. Mass spectra were calibrated externally using a mixture of peptides comprising Angiotensin I, adrenocorticotropic hormone (ACTH) fragments containing residues 1-17, 7-38 and 18-39, and a synthetic hemagglutinin antigen derived peptide. Mass spectra were processed using the Data Analysis v3.4 software (Bruker Daltonics, Billerica, MA, USA) and recalibrated internally utilizing identified peptide ions in each spectrum derived from the viral proteins. Mass lists were exported as tab-delimited files. Mass accuracies of between 0.1 to 1 ppm are routinely achieved for all ions detected with mass resolutions (FWHM) exceeding 100,000.

## Availability and Requirements

**Project name: **FluTyper

Project home page:

http://www.cancerresearch.unsw.edu.au/CRCWeb.nsf/page/flutyper

**Operating system: **Windows, Linux

**Programming language: **C++

**License: **Free for non-commercial use. Source code available upon request.

## Authors' contributions

JWHW designed, developed and implemented the algorithm and wrote the manuscript. ABS designed the algorithm, carried out the mass spectrometry experiments, prepared the virus digest and drafted the manuscript. KMD conceived the project, participated in its design and coordination and drafted the manuscript. All authors read and approved the final manuscript.

## Supplementary Material

Additional file 1 Zipped file containing the raw mass spectra used for testing FluTyper. Click here for file

Additional file 2 Screenshot of the input web interface for FluTyper. Click here for file

Additional file 3 Description of parameters used in FluTyper Click here for file

Additional file 4 **FluTyper HTML web output for influenza type A (H3N2) strain A/Brisbane/10/2007 shown in Figure**3D. Click here for file

Additional file 5 **FluTyper output on the analysis of the mass spectrum of whole virus digest of type A influenza (H3N2) strain A/Brisbane/10/2007 shown in Figure**3B. Click here for file
