# Supplementary figures and images for: FluTyper-an algorithm for automated typing and subtyping of the influenza virus from high resolution mass spectral data
Source: BMC Bioinformatics. 2010 May 19;11:266. doi: 10.1186/1471-2105-11-266 (PMC3098065; doi:10.1186/1471-2105-11-266)

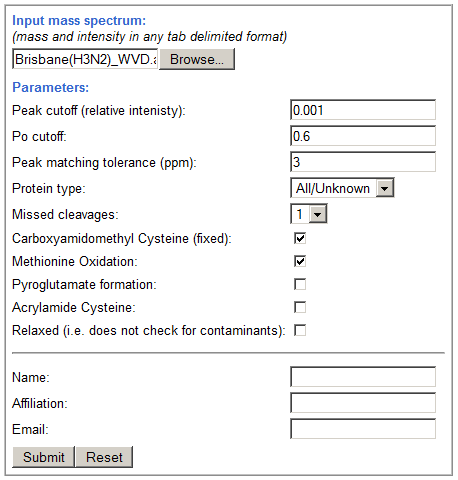

Supplement: Additional file 2 — Screenshot of the input web interface for FluTyper. [file 1471-2105-11-266-S2.TIFF]

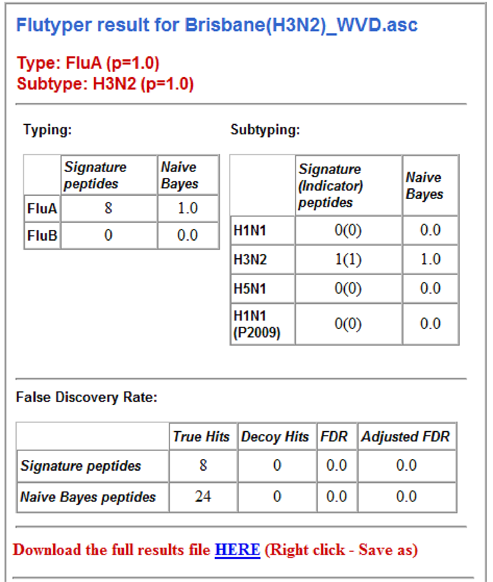

Supplement: Additional file 4 — FluTyper HTML web output for influenza type A (H3N2) strain A/Brisbane/10/2007 shown in Figure3D. [file 1471-2105-11-266-S4.TIFF]
