# Supplementary material for: FluTyper-an algorithm for automated typing and subtyping of the influenza virus from high resolution mass spectral data
Source: BMC Bioinformatics. 2010 May 19;11:266. doi: 10.1186/1471-2105-11-266 (PMC3098065; doi:10.1186/1471-2105-11-266)
Supplement: Additional file 3 — Description of parameters used in FluTyper [file 1471-2105-11-266-S3.DOC]

**Additional Table 3.** Description of parameters used in FluTyper

| Parameters | Description | Default value |
| --- | --- | --- |
| Peak cutoff (relative intensity) | Cutoff at which to filter low intensity peaks | 0.001 |
| P_o_ cutoff | Cutoff at which to ignore masses that have a probability P_o_ < P_o,cutoff_ across all types or subtypes | 0.6 |
| Peak matching tolerance (ppm) | Error tolerance for matching a theoretical mass to a peak in a mass spectrum | 3 |
| Missed cleavages | Maximum number of missed tryptic cleavages to anticipate | 1 |
| Possible modifications | CAM-Cys, Met-Ox, Pyro-Glu, PAM-Cys | CAM-Cys, Met-Ox |
| Relaxed | If checked, the uniqueness of masses are not evaluated which can lead to increased false positive identifications. This option may be useful if high-resolution (i.e. < 5 ppm) cannot be achieve by the mass spectrometer being used. | 0 |
| Name/Affiliation/Email | These are optional |  |
